# Supplementary material for: Uncovering Phytotoxic Compounds Produced by Colletotrichum spp. Involved in Legume Diseases Using an OSMAC–Metabolomics Approach
Source: J Fungi (Basel). 2023 May 25;9(6):610. doi: 10.3390/jof9060610 (PMC10302859; doi:10.3390/jof9060610)
Supplement: Supplementary file 1 [file jof-09-00610-s001.zip › jof-2394021-supplementary.pdf]

Journal of Fungi

MDPI

Original Article

Uncovering Phytotoxic Compounds Produced by *Colletotrichum* spp. Involved in Legume Diseases Using an OSMAC–Metabolomics Approach

Pierluigi Reveglia [1,†], Francisco J. Agudo-Jurado [1,†], Eleonora Barilli [1], Marco Masi [2], Antonio Evidente [2,3] and Diego Rubiales [1,*]

1 Institute for Sustainable Agriculture, CSIC, 14004 Cordoba, Spain
2 Department of Chemical Sciences, University of Naples Federico II (UNINA), 80126 Naples, Italy
3 Institute of Sciences of Food Production, National Research Council (ISPA-CNR), 70125 Bari, Italy
* Correspondence: diego.rubiales@ias.csic.es
† These authors contributed equally to this work.

Supplementary material

Figure S1. m/z distribution diagrams and Base Peak Chromatograms of *C. truncatum* isolate C428 (from lentil) extracts. (a) PDA extract; (b) PDB extract; (c) Rice extract; (d) Richard extract.

Figure S2. m/z distribution diagrams and Base Peak Chromatograms of *C. trucatum* isolate C431 (from soybean) extracts. (a) PDA extract; (b) PDB extract; (c) Rice extract; (d) Richard extract.

Figure S3. m/z distribution diagrams and Base Peak Chromatograms of *C. trifolii* isolate C436 (from clover extracts. (a) PDA extract; (b) PDB extract; (c) Rice extract; (d) Richard extract.

Figure S4. Structure of: validated metabolites with pure standards (level A; red); putatively identified and produced by *Colletotrichum* spp. (level B(i), blue); putatively identified and produced by other fungal species (level B(ii), black); Structures of Curvupallide A and B (Level C(i), brown).

Table S1. Parameters Metaboanalyst 5.0 for LC-MS spectra processing.

Table S2. Features lists transformation and scaling for PLS – DA analysis. Q2 and R2 values of PLS – DA models in cross validation.

Table S3. Metabolites dereplicated by targeted and untargeted metabolomics analysis, organised according to identification level.

Table S4. Metabolites dereplicated by targeted and untargeted metabolomics analysis, organised according to identification level.

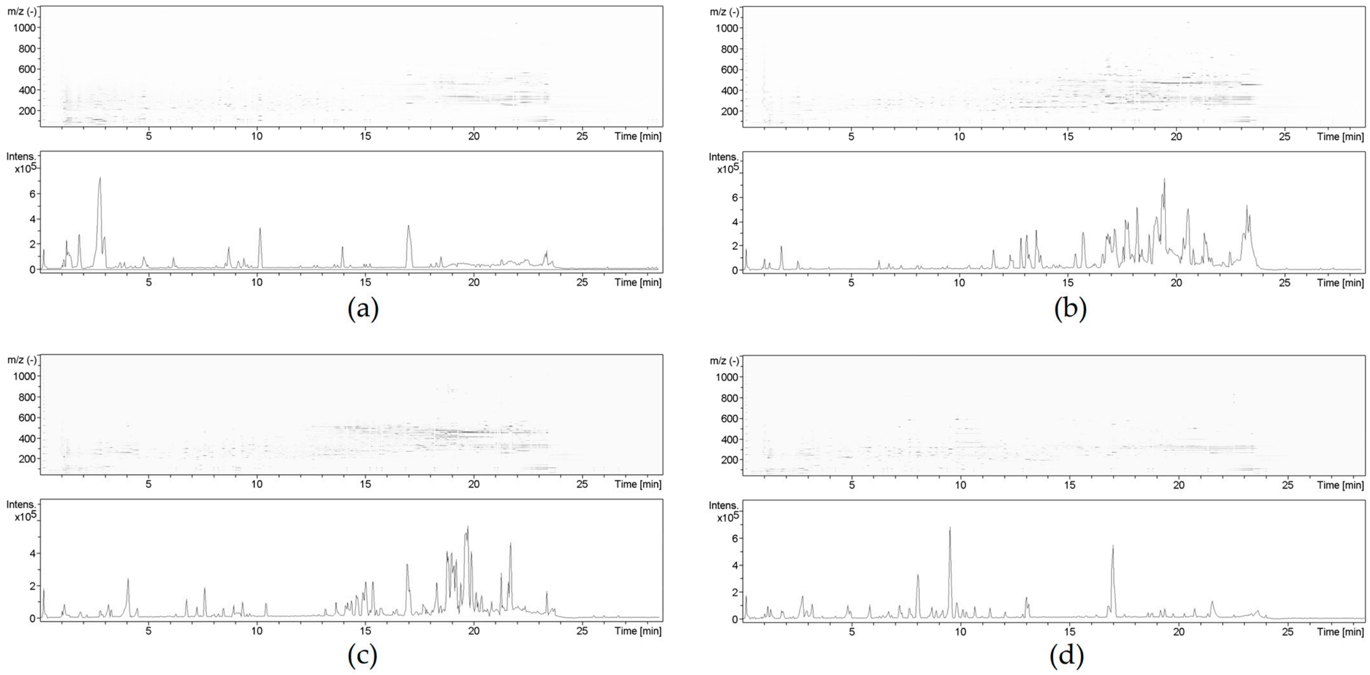

Figure S1. m/z distribution diagrams and Base Peak Chromatograms of *C. truncatum* isolate C428 (from lentil) extracts. (a) PDA extract; (b) PDB extract; (c) Rice extract; (d) Richard extract.

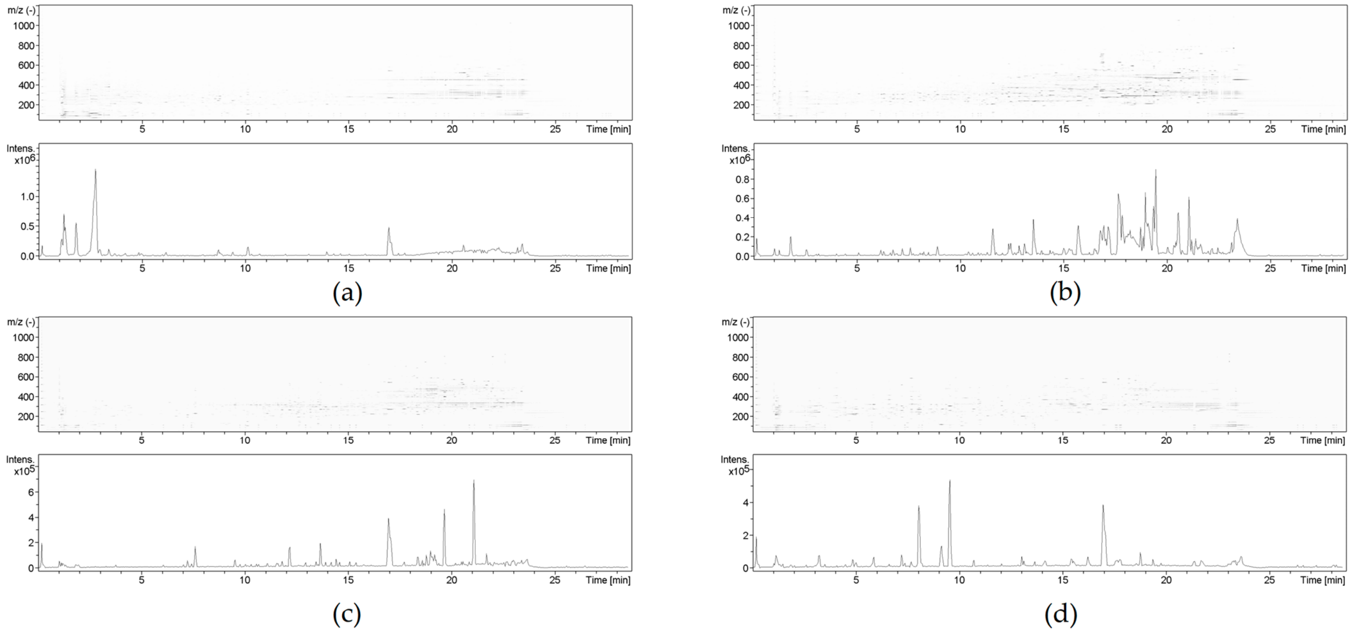

Figure S2. m/z distribution diagrams and Base Peak Chromatograms of *C. trucatum* isolate C431 (from soybean) extracts. (a) PDA extract; (b) PDB extract; (c) Rice extract; (d) Richard extract.

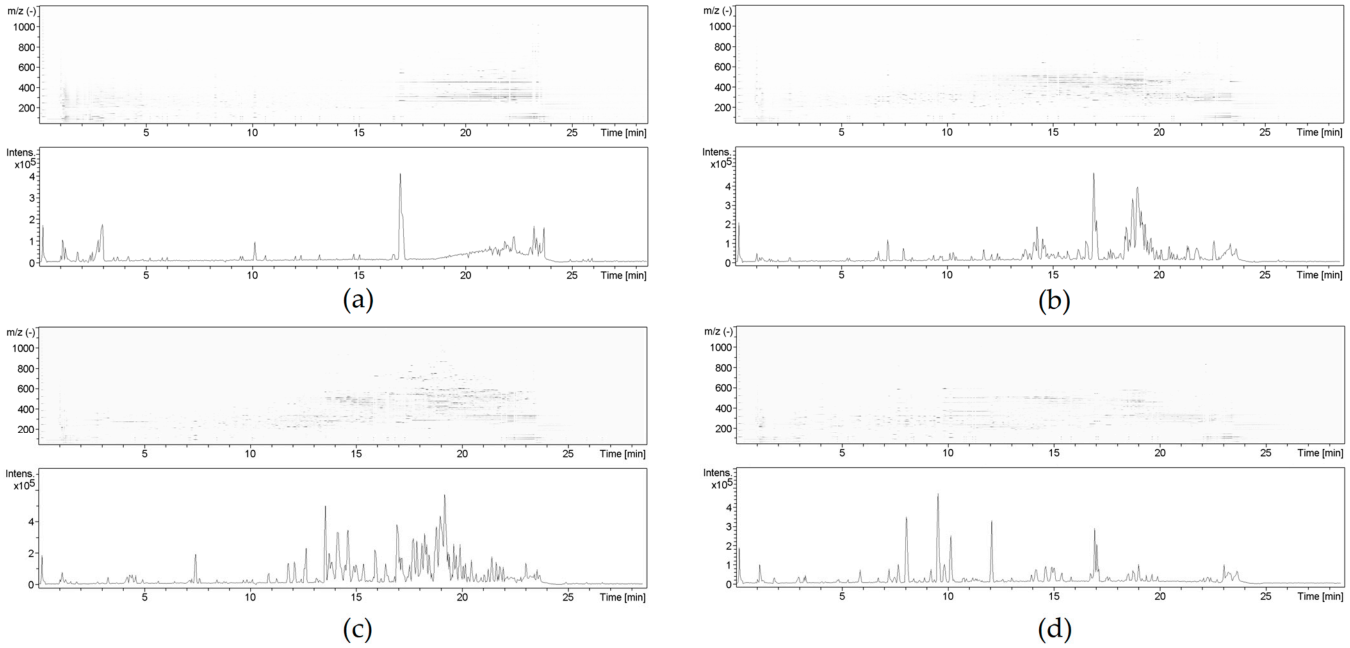

Figure S3. m/z distribution diagrams and Base Peak Chromatograms of *C. trifolii* isolate C436 (from clover) extracts. (a) PDA extract; (b) PDB extract; (c) Rice extract; (d) Richard extract.

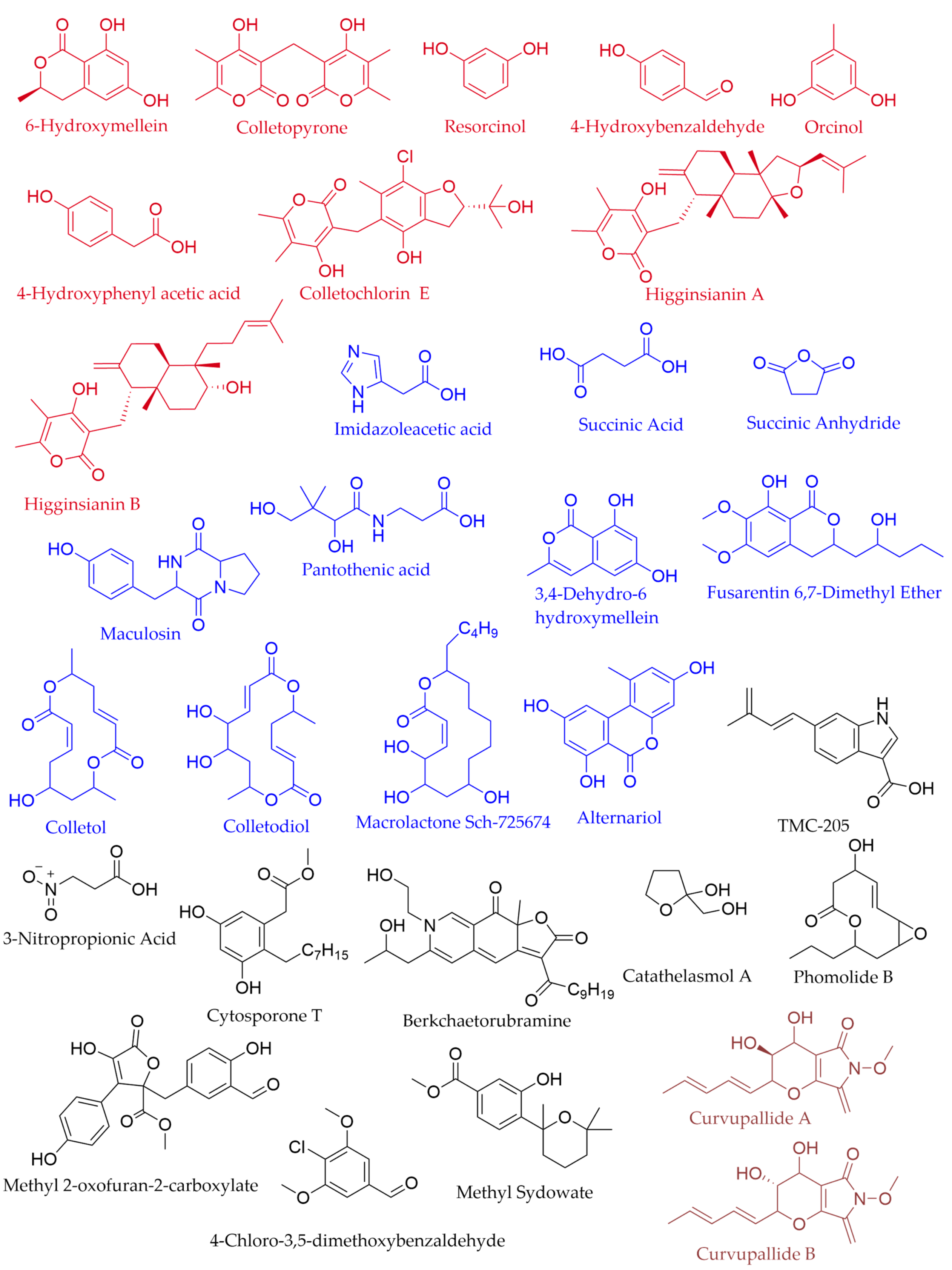

Figure S4. Structure of: validated metabolites with pure standards (level A; red); putatively identified and produced by *Colletotrichum* spp. (level B(i), blue); putatively identified and produced by other fungal species (level B(ii), black); Structures of Curvupallide A and B (level C(i), brown).

Table S1. Parameters Metaboanalyst 5.0 for LC-MS spectra processing.

**Platform selection**	**Parameters**
UPLC-Q/TOF	Auto-Optimized
**Peak Picking**	**Parameters**
Method	centWave
min_peakwidth	5.0
max_peakwidth	20.0
ppm	15 ppm
mzdiff	0.01
snthresh	6.0
noise	1000.0
prefilter	3.0
value_of_prefilter	100.0
**Peak Alignment**	**Parameters**
Method	obiwarp
Bandwidth	30.0
minFraction	0.8
minSamples	1
maxFeatures	100
integrate	1
extra	1
span	0.25
profStep	1
**Peak Alignment**	**Parameters**
Polarity	Negative
Adducts	$[M]^-$;$[M-H]^-$; $[M-2H]^-$; $[M-3H]^-$; $[M-H+Cl]^{2-}$; $[M+Cl]^-$; $[M+2Cl]^{2-}$;$[2M-H]^-$
Perc_fwhm	0.6
Mz_abs_iso	0.005
Max_charge	2
Corr_eic_th	0.85
Mz_abs_add	0.001
Contaminant Removal	Yes
Blank Subtraction	Yes

Table S2. Features lists transformation and scaling for PLS – DA analysis. Q2 and R2 values of PLS – DA models in cross validation.

**Cultural media extracts**	**Transformation**	**Scaling**	**Q2 value**	**R2 value**
PDA	Logarithmic	Pareto scaling	0.8331*	0.9900*
PDB	Logarithmic	Range scaling	0.9106**	0.9997**
Rice	Logarithmic	Pareto scaling	0.9501**	0.9998**
Richard	Cube root	Autoscaling	0.7625***	0.9967***

*values using the top 4 components; ** values using the top 5 components; ***values using the top 3 components.

Table S3. Metabolites dereplicated by targeted and untargeted metabolomics analysis, organised according to identification level.

**Putative metabolite name**	**Biosynthetic origin and Class of compound**	**Molecular formula**	**Ret. Time**	**ES(-) Theor. m/z**	**ES(-) Found m/z**	**m/z error (mDa)**	**MS/MS**	**ID Levels (A – D)***	**Databases Code****
6-hydroxymellein	Polyketides_ Isocoumarine	$C_{10}H_{10}O_4$	12.58	193.0501 [M-H]-	193.0495 [M-H]-	0.6	149.0601; 131.0498; 105.0715	A	CNP0395266 NPA028477
Colletopyrone	Polyketides_Cyclic polyketides	$C_{15}H_{16}O_6$	18.24	291.0869 [M-H]-	291.0904 [M-H]-	-3.5	224.0162; 139.0394; 95.0505	A	CNP0267088 Q82653372
Resorcinol	Shikimates and Phenylpropanoids_Simple phenol	$C_6H_6O_2$	10.11	109.0289 [M-H]-	109.0284 [M-H]-	0.5	93.0330; 81.0331	A	CNP0414137 Q82653372
4-Hydroxybenzaldehyde	Shikimates and Phenylpropanoids_Simple phenolic acids	$C_7H_6O_2$	9.12	121.0289 [M-H]-	121.0291 [M-H]-	-0.2	108.0200; 92.0271; 65.0407	A	CNP0230702 NPA032546
Orcinol	Shikimates and Phenylpropanoids_Simple phenol	$C_7H_8O_2$	4.76	123.0446 [M-H]-	123.0446 [M-H]-	-	109.0292; 108.0190; 91.0180; 81.0350	A	CNP0300146 NPA032537
4-Hydroxyphenylacetic acid	Shikimates and Phenylpropanoids_Simple phenolic acids	$C_8H_8O_3$	8.97	151.0395 [M-H]-	151.0390 [M-H]-	0.5	118.8785; 109.0283; 107.049; 93.0333	A	CNP0212603 NPA032547

Colletochlorin E	Terpenoids& Polyketides_Tetraketide meroterpenoids	$C_{20}H_{23}ClO_6$	18.72	393.1104 [M-H]-	393.1105 [M-H]-	-0.1	241.0638; 151.0398; 139.0400; 95.0504	A	-
Higginsianin A	Terpenoids& Polyketides_Triketide meroterpenoids	$C_{27}H_{38}O_4$	22.50	425.2692 [M-H]-	425.2686 [M-H]-	0.6	325.1839; 265.1463; 265.1478; 223.0281	A	CNP0152211 NPA021815
Higginsianin B	Terpenoids& Polyketides_Triketide meroterpenoids	$C_{27}H_{40}O_4$	22.66	427.2848 [M-H]-	427.2843 [M-H]-	0.5	357.2645; 315.2527; 283.2276; 155.0351	A	CNP0073478 NPA021814
Imidazoleacetic acid	Alkaloids_Imidazole alkaloids	$C_5H_6N_2O_2$	3.15	125.0351 [M-H]-	125.0350 [M-H]-	0.1	81.04434; 71.6317; 97.0401	B(i)	CNP0253943
Maculosin	Amino acids and Peptides_Dipeptides	$C_{14}H_{16}N_2O_3$	7.22	259.1083 [M-H]-	259.1090 [M-H]-	-0.5	98.0251; 121.0293; 124.0398	B(i)	CNP0320469 NPA007345
Succinic Acid	Fatty Acids_ Dicarboxylic acids	$C_4H_6O_4$	2.74	117.0188 [M-H]-	117.0190 [M-H]-	-0.2	99.00871; 73.0295	B(i)	Q213050
Succinic Anhydride	Fatty Acids_ Anhydride of dicarboxylic acid	$C_4H_4O_3$	2.76	99.0082 [M-H]-	99.0091 [M-H]-	-0.9	73.0138; 59.0155	B(i)	CNP0079889 Q417847
Pantothenic acid	Fatty acids_ Beta amino acids and derivatives	$C_9H_{17}NO_5$	4.94	218.1028 [M-H]-	218.1038 [M-H]-	-1	88.0413; 99.0459;	B(i)	CNP0153142

							146.0810,<br>71.01495		
3,4-Dehydro-6-hydroxymellein	Polyketides_ Isocoumarine	$C_{10}H_8O_4$	16.10	191.0344<br>$[M-H]^-$	191.0352<br>$[M-H]^-$	-0.8	149.0234;<br>147.0441;<br>123.0439;<br>87.0458	B(i)	CNP0389619<br>Q27105447
Fusarentin 6,7-dimethyl ether	Polyketides_ Isocoumarine	$C_{16}H_{22}O_6$	15.70	309.1338<br>$[M-H]^-$	309.1323<br>$[M-H]^-$	1.5	265.1440;<br>253.1075;<br>233.1208;<br>125.0243	B(i)	CNP0219353<br>Q27138491
Colletol	Polyketides_Macrolides	$C_{14}H_{20}O_5$	15.2	267.1235<br>$[M-H]^-$	267.1240<br>$[M-H]^-$	-0.5	223.1343;<br>213.1496;<br>121.0296	B(i)	CNP0157857<br>NPA002061
Colletodiol	Polyketides_Macrolides	$C_{14}H_{20}O_6$	13.7	283.1182<br>$[M-H]^-$	283.1187<br>$[M-H]^-$	-0.5	111.0822;<br>253.1077;<br>207.1020;<br>239.1284	B(i)	Q27896955
Macrolactone Sch-725674	Polyketides_Macrolides	$C_{18}H_{32}O_5$	18.4	327.2172<br>$[M-H]^-$	327.2179<br>$[M-H]^-$	-0.7	209.1179;<br>227.1282;<br>293.1770	B(i)	CNP0221935<br>NPA008159
Alternariol	Polyketides_Isocoumarine	$C_{14}H_{10}O_5$	17.53	257.0450<br>$[M-H]^-$	257.0457<br>$[M-H]^-$	-0.7	111.0087;<br>213.0557;<br>68.9974	B(i)	CNP0184929<br>NPA020438
TMC-205	Alkaloids_Simple indole alkaloids	$C_{14}H_{13}NO_2$	4.83	226.0868<br>$[M-H]^-$	226.0.884<br>$[M-H]^-$	-1.6	121.0294;<br>135.0440<br>167.0367	B(ii)	CNP0402436<br>NPA017297

3-Nitropropionic Acid	Fatty Acids_Oxo fatty acids	$C_3H_5NO_4$	2.73	118.0140 $[M-H]^-$	118.0145 $[M-H]^-$	-0.5	99.0075; 73.0281; 61.9896	B(ii)	CNP0439550 NPA007876
Cytosporone T	Polyketides_Aromatic polyketides	$C_{17}H_{26}O_4$	19.30	293.1752 $[M-H]^-$	293.1762 $[M-H]^-$	-1	275.16412; 221.15503; 236.10591; 83.05134; 149.09648	B(ii)	CNP0264849 NPA018477
Berkchaetorubramine	Polyketides_Azaphilones	$C_{27}H_{37}NO_6$	20.34	470.2543 $[M-H]^-$	470.2549 $[M-H]^-$	-0.6	414.1930; 301.1450; 251.1654; 111.0095	B(ii)	CNP0357282 NPA004011
Catathelasmol A	Polyketides_furans	$C_5H_{10}O_3$	4.58	117.0552 $[M-H]^-$	117.0553 $[M-H]^-$	-0.1	101.0246; 85.0300; 73.0304; 68.0517	B(ii)	CNP0357577 NPA010903
Phomolide B	Polyketides_Macrolides	$C_{12}H_{18}O_4$	16.08	225.1127 $[M-H]^-$	225.1134 $[M-H]^-$	-0.7	137.0970; 89.0248; 151.0764; 121.0295; 127.0764; 195.0647; 68.9970	B(ii)	CNP0206816 NPA005349

Methyl 2-[(3-formyl-4-hydroxyphenyl)methyl]-4-hydroxy-3-(4-hydroxyphenyl)-5-oxo-2,5-dihydrofuran-2-carboxylate	Shikimates and Phenylpropanoids_Lignans	$C_{20}H_{16}O_8$	16.5	351.0869 $[M-H]^-$	383.0772 $[M-H]^-$	-0.5	339.0872; 157.0136; 95.0550; 89.0252	B(ii)	CNP0354744 NPA014159
4-hloro-3,5-dimethoxybenzaldehyde	Shikimates and Phenylpropanoids_Simple phenolic acids	$C_9H_9ClO_3$	3.02	199.0162 $[M-H]^-$	199.0127 $[M-H]^-$	3.5	135.04427; 121.0297; 93.03511	B(ii)	CNP0247714 NPA002948
(+)-Methyl Sydowate	Terpenoids_Sesquiterpenoids	$C_{16}H_{22}O_4$	17.00	277.1439 $[M-H]^-$	277.1446 $[M-H]^-$	-0.7	134.0379; 127.1134; 121.0300; 111.0092; 77.0408	B(ii)	CNP0134087 NPA010996
Curvupallide A or B	Polyketides_ Isoindolazinfuranones	$C_{14}H_{17}NO_5$	10.41	278.1028 $[M-H]^-$	278.1035 $[M-H]^-$	-0.7	205.0868; 248.0924; 163.0757; 178.0858; 124.0402; 135.0445	C(i)	NPA004814/ NPA015714
3-Hydroxydodecanedioic acid; Dibutyl malate;	Fatty Acids_ Dicarboxylic acids	$C_{12}H_{22}O_5$	11.0	245.1389 $[M-H]^-$	245.1395 $[M-H]^-$	-0.6	173.0810; 111.0818; 141.0919; 103.0509	C(i)	-
Fumaric acid; Maleic acid;	Fatty Acids_ Dicarboxylic acids	$C_4H_4O_4$	2.56	115.0031 $[M-H]^-$	115.0033 $[M-H]^-$	-0.2	87.0091; 71.0139	C(i)	-

Dihydroxybutyric acid	Fatty Acids_Dihydroxy Fatty acid	$C_4H_8O_4$	1.26	119.0344 [M-H]-	119.0346 [M-H]-	-0.2	71.01505; 86.02505; 101.02489	C(i)	-
Hydroxybutyric acid	Fatty Acids_Hydroxy Fatty acid	$C_4H_8O_3$	2.95	103.0395 [M-H]-	103.0402 [M-H]-	-0.7	57.0366; 74.0233; 85.02810; 101.0232	C(i)	-
Trihydroxy octadecenoic acid	Fatty Acids_Long Chain Fatty Acids	$C_{18}H_{34}O_5$	17.54	329.2328 [M-H]-	329.2335 [M-H]-	0.7	229.14413; 211.1333; 171.1020; 162.02217	C(i)	-
p-Cresol; m-Cresol; o-Cresol; Benzyl alcohol; Anisole;	Shikimates and Phenylpropanoids_Simple phenol	C_7H_8O	10.1	107.0497 [M-H]-	107.0504 [M-H]-	-0.7	85.0304; 79.0540; 65.0380	C(i)	-
2,5-Dimethylfuran; 2-Ethylfuran	Polyketides_Furans	C_6H_8O	7.41	95.0497 [M-H]-	95.0508 [M-H]-	-1.1	82.0305; 68.9968; 55.0207	C(i)	-
Putative jasmonic acid derivative	Fatty acids_Octadecanoids_Jasmonic acids	$C_{12}H_{20}O_3$	19.0	211.1334 [M-H]-	211.1340 [M-H]-	-0.6	111.0091; 151.0762; 149. 0458	C(ii)	-
Putative colletotryptin	Alkaloids_Tryptophan alkaloids	$C_{20}H_{22}N_2O_5$	14.1	369.1450 [M-H]-	369.1457 [M-H]-	-0.7	325.1548; 255.0776; 221.0931	C(ii)	-
Putative glycosylamine	Carbohydrates and carbohydrate conjugate	$C_{10}H_{14}N_2O_5$	3.21	241.0824 [M-H]-	241.0832 [M-H]-	-0.8	223.0721; 197.0935; 144.0658; 145.0501	C(ii)	-

Putative hypoxanthine	Alkaloids _Hypoxanthines	$C_8H_{11}N_5O_3$	4.83	225.0862 $[M]^-$	225.0885 $[M]^-$	-2.3	165.0653; 88.0413; 82.0306; 131.0347; 113.0606	C(ii)	-
Putative colletopeptide	Cyclic peptides _ Depsipeptides	$C_{25}H_{29}N_5O_7$	14.14	510.1989 $[M-H]^-$	510.1978 $[M-H]^-$	1.1	494.2041; 448.1982; 281.1398; 162.0547	C(ii)	-
Putative colletopeptide	Cyclic peptides _ Depsipeptides	$C_{26}H_{29}N_3O_6$	18.31	478.1978 $[M-H]^-$	478.2036 $[M-H]^-$	5.0	448.1977; 281.1394; 267.1588	C(ii)	-
Putative colletopeptide	Cyclic peptides _ Depsipeptides	$C_{28}H_{42}N_4O_5$	21.70	513.3077 $[M-H]^-$	513.3071 $[M-H]^-$	0.6	423.2756; 358.2028; 297.2437	C(ii)	
Putative colletopeptide	Cyclic peptides _ Depsipeptides	$C_{29}H_{45}N_5O_5$	17.9	542.3342 $[M-H]^-$	542.3335 $[M-H]^-$	0.7	527.3226; 515.3228; 501.3428; 499.3272; 489.2980	C(ii)	-
Putative colletopeptide	Cyclic peptides _ Depsipeptides	$C_{31}H_{33}N_3O_7S$	9.82	590.1961 $[M-H]^-$	590.1953 $[M-H]^-$	0.8	502.1041; 444.0982; 275.1383; 178.0865	C(ii)	-
Putative benzene derivative	Benzenoids _Benzene and substituted derivatives	$C_{20}H_{18}O_3$	9.51	305.1178 $[M-H]^-$	305.1144 $[M-H]^-$	3.4	261.1863; 305.1752; 155.0141;	C(ii)	-

							111.0094		
Putative O-glycosil compound	Carbohydrates and carbohydrate conjugate_O-glycosil compounds	$C_{12}H_{18}O_9$	4.77	305.0873 $[M-H]^-$	305.0880 $[M-H]^-$	-0.7	306.8868; 225.0893; 181.1003; 131.0336 82.0305	C(ii)	-
Putative O-glycosil compound	Carbohydrates and carbohydrate conjugate_O-glycosil compounds	$C_{16}H_{28}O_6$	11.5	315.1808 $[M-H]^-$	315.1812 $[M-H]^-$	-0.4	283.1550; 211.0609; 68.9967	C(ii)	-
Putative phenolic glycoside	Carbohydrates and carbohydrate conjugate_Phenolic glycosides	$C_{15}H_{19}NO_8$	9.78	340.1032 $[M-H]^-$	340.1035 $[M-H]^-$	-0.3	294.0978; 221.0702; 192.0661; 138.0202; 108.0457	C(ii)	-
Putative mycosporine	Amino acids and Peptides_ Mycosporine derivatives	$C_{15}H_{17}NO_6$	6.16	306.0977 $[M-H]^-$	306.0984 $[M-H]^-$	-0.7	260.1046; 121.02924; 124.0404	C(ii)	
Putative tricarboxylic acid derivative	Carboxylic acid and derivatives_tricarboxylic acid and derivatives	$C_{12}H_{20}O_7$	17.54	275.1131 $[M-H]^-$	275.1137 $[M-H]^-$	-0.6	238.0693; 214.0169; 129.0194; 112.9852	C(ii)	-
Putative cyclic fatty acyl glycosides	Fatty Acids_ Fatty acyl glycosides	$C_{28}H_{48}O_9$	21.31	527.3220 $[M-H]^-$	527.3223 $[M-H]^-$	-0.3	470.2545; 400.2484; 348.2541;	C(ii)	-

							279.2345; 148.9299; 263.0919; 189.0532		
Putative isocoumarine	Polyketides_ Isocoumarine	$C_{11}H_{11}ClO_5$	2.76	257.0217 [M-H]-	257.0252 [M-H]-	-3.5	103.0389; 89.02373; 73.03042	C(ii)	-
Putative aromatic polyketide	Polyketides_Aromatic Polyketides	$C_{16}H_{16}O_5$	4.47	287.0919 [M-H]-	287.0885 [M-H]-	3.4	107.05028; 96.95872; 79.95595	C(ii)	-
Putative polycyclic aromatic polyketides	Polyketides_Polycyclic aromatic polyketides	$C_{18}H_{12}O_8$	16.80	355.0454 [M-H]-	355.0459 [M-H]-	-0.5	341.0662; 271.1446; 244.1556	C(ii)	-
Putative zearalenones	Polyketides_Zearalenones	$C_{16}H_{30}O_6$	14.20	317.1964 [M-H]-	317.1967 [M-H]-	-0.3	148.92999; 263.09186; 189.0532; 116.0503	C(ii)	-
Patative β amino acid derivative	Beta amino acids and derivatives	$C_9H_{12}N_2O_4$	3.67	211.0718 [M-H]-	211.0727 [M-H]-	-0.9	167.0816; 114.0196; 102.9327; 70.0304	C(ii)	
Putative phenolic acid	Shikimates and Phenylpropanoids_ Phenolic acids (C6-C1)	$C_{17}H_{17}NO_3$	4.76	282.1130 [M-H]-	282.1096 [M-H]-	3.4	192.0671; 92.05122; 148.0767	C(ii)	-

Putative phenolic acid	Shikimates and Phenylpropanoids_ Simple phenolic acids	$C_9H_9NO_4$	7.23	194.0453 $[M-H]^-$	194.0463 $[M-H]^-$	-1	150.0561; 137.0240; 108.0460; 107.0377	C(ii)	-
Putative phenylethanoid	Shikimates and Phenylpropanoids_Phenylethanoids	$C_{21}H_{32}O_{12}$	18.75	476.1893 $[M-H]^-$	476.1926 $[M-H]^-$	-3.3	460.1980; 430.1876; 237.1130; 149.0462	C(ii)	-
Putative anisol	Benzenoids _Anisoles	$C_{24}H_{31}NO_8$	19.32	460.1971 $[M-H]^-$	460.1978 $[M-H]^-$	-0.7	414.1924; 384.1817; 366.1709; 309.1538; 88.0047	C(ii)	-
Putative xanthone	Shikimates and Phenylpropanoids_Xanthones	$C_{28}H_{30}O_6$	19.60	461.1964 $[M-H]^-$	461.2009 $[M-H]^-$	-4.5	415.1001; 253.0480; 275.1405	C(ii)	-
Putative triketide meroterpenoid	Terpenoids& Polyketides_Triketide meroterpenoids	$C_{28}H_{32}O_9$	14.20	511.1968 $[M-H]^-$	511.2009 $[M-H]^-$	-4.1	421.1874; 403.1775; 375.1814; 369.1350; 299.1290	C(ii)	-
Putative terpene glycoside	Terpene glycosides	$C_{24}H_{32}NO_{10}$	14.94	494.2026 $[M]^-$	494.2030 $[M]^-$	-0.4	416.175; 369.1549; 323.1500	C(ii)	
Putative fusarin	Terpenoids_Sesquiterpenoids	$C_{23}H_{30}NO_7$	18.82	431.1944 $[M-H]^-$	431.1905 $[M-H]^-$	3.9	416.1710; 402.1710;	C(ii)	-

							389.1598; 324.1452; 303.0860		
Putative fusarin	Terpenoids_Sesquiterpenoids	$C_{23}H_{29}NO_6$	19.01	414.1917 [M-H]-	414.1924 [M-H]-	-0.7	387.1815; 301.1440; 352.21173; 213.12711	C(ii)	-
Putative fusarin	Terpenoids_Sesquiterpenoids	$C_{23}H_{29}NO_7$	18.56	430.1866 [M-H]-	430.1873 [M-H]-	0.7	400.1759; 357.1609; 320.1550; 151.0766; 139.0042; 138.0199	C(ii)	-
Putative fusarin	Terpenoids_Sesquiterpenoids	$C_{23}H_{29}NO_8$	16.41	446.1815 [M-H]-	446.1821 [M-H]-	-0.6	414.1925; 400.1767; 241.1453	C(ii)	-
Putative fusarin	Terpenoids_Sesquiterpenoids	$C_{23}H_{29}NO_9$	15.50	462.1764 [M-H]-	462.1770 [M-H]-	-0.6	448.1987; 416.1725; 323.1037; 307.1085; 196.0982	C(ii)	-
Putative fusarin	Terpenoids_Sesquiterpenoids	$C_{23}H_{31}NO_7$	18.24	432.2022 [M-H]-	432.2029 [M-H]-	-0.7	416.1710; 402.1710; 389.1598; 324.1452; 303.087	C(ii)	-

Putative fusarin	Terpenoids_Sesquiterpenoids	$C_{23}H_{31}NO_8$	15.12	448.1971 $[M-H]^-$	448.1977 $[M-H]^-$	-0.6	432.1654; 416.1712; 167.0717; 155.0461	C(ii)	-
Unknown	-	$C_{20}H_{32}O_9$	18.82	415.1968 $[M-H]^-$	415.1959 $[M-H]^-$	0.9	-	D	-
Unknown	-	$C_{17}H_{24}O_6$	15.50	323.1495 $[M-H]^-$	323.1502 $[M-H]^-$	-0.7	-	D	-
Unknown	-	$C_7H_{14}N_2O_4S_2$	7.42	253.0317 $[M-H]^-$	253.0341 $[M-H]^-$	-2.4	-	D	-
Unknown	-	$C_{16}H_{24}O_6$	15.03	311.1495 $[M-H]^-$	311.1499 $[M-H]^-$	-0.4	-	D	-
Unknown	-	$C_{22}H_{36}O_{13}$	16.63	508.2155 $[M]^-$	508.2187 $[M]^-$	-3.2	-	D	-
Unknown	-	$C_{26}H_{36}O_6$	16.50	524.2137 $[M-H]^-$	524.2137 $[M-H]^-$	-	-	D	-
Unknown	-	$C_{27}H_{30}O_6$	15.17	449.1964 $[M-H]^-$	449.2011 $[M-H]^-$	-4.7	-	D	-

Unknown	-	$C_{27}H_{35}NO_6$	19.4	468.2386 [M-H]$^-$	468.2386 [M-H]$^-$	-	D	-
Unknown	-	-	3.29	-	312.0872	-	D	-
Unknown	-	-	23.5	-	600.1905	-	D	-
Unknown	-	-	0.17	-	452.9227	-	D	-
Unknown	-	-	14.98	-	495.2018	-	D	-

*Confidence levels: Level A: validate metabolite. Comparison with pure standard analysed in the same experimental LC-MS/MS condition; Level B(i): Putative identification using taxonomical information. Confident match based on MS/MS data with reference standard of selected spectral libraries in MSDIAL/MSFINDER analysis (Match Score ≥ 8) and/or complete match of *in silico* fragmentation of MS/MS data of compounds restricting interrogation to *Colletrotrichum* species using MetFrag; Level B(ii): Putative identification. Complete match of *in silico* fragmentation of MS/MS, using MetFrag data restricting interrogation to fungal metabolites. Level C(i): Tentative structure. MS/MS data in agreement with different structural isomers of reference standards of selected database in MSDIAL/MSFINDER belonging to the same natural compound class; Level C(ii): Compound Class. Partial match of MS/MS data on the spectral libraries used in MSDIAL/MSFIDER (6 ≤ Match Score < 8) with specific metabolites class and/or MS/MS data in agreement with a specific class of fungal metabolites according to *in silico* fragmentation using MetFrag; Level D: Only molecular formula and/or High-resolution mass.

**Databases identifiers: ID starting with CNPXXX are Coconunt ID: https://coconut.naturalproducts.net/; ID starting with NPAXXX are Natural Product Atlas ID: https://www.npatlas.org/; ID starting with QXXX are Lotus ID: https://lotus.naturalproducts.net/.

Table S4. Metabolites dereplicated by targeted and untargeted metabolomics analysis, organised according to identification level.

		**Richard**					**PDA**					**PDB**					**Rice**				
**Fungal species**	**Legume species tested**	**N[a]**	**DA[a]**	**NR[a]**	**%DS 1 mg/ml**	**%DS 2mg/ml**	**N**	**DA**	**NR**	**%DS 1 mg/ml**	**%DS 2 mg/ml**	**N**	**DA**	**NR**	**%DS 1 mg/ml**	**%DS 2 mg/ml**	**N**	**DA**	**NR**	**%DS 1 mg/ml**	**%DS 2 mg/ml**
C.	Lentil	+	++	+	100	100	+	++	+	89.7±14.5	100	+	++	+	72.8±19.5	84.7±14.2	+	+	-	23.7±9.6	51.8±16*
truncat	Soybean	-	+	+	11.9±5	12.2±4.8	+	-	-	4.4±1.6	4.6±1.9	+	-	-	3.4±0.9	3.6±1.1	+	-	-	7.1±1.5	8.5±1.3
um	Faba bean	+	-	-	4.3±2.5	9±2.1*	+	-	-	4.3±2.1	2.9±1	+	+	-	2.4±1.6	6.4±2.4*	+	-	-	11.6±2.3	19±4.8*
isolate	Pea	-	+	+	20.2±8.3	26.5±4.8	+	+	-	4.4±2.3	5.9±2.7	-	+	-	4.2±2.3	35±14*	+	+	+	9.3±4.2	8.4±5.9
C428	Red clover	-	+	+	35.5±10.5	80.6±16*	-	+	-	31±8.9	34.4±11.4	-	+	+	27.1±6.5	28.7±2.8	-	+	+	42±13.4	72.1±18.5*
	White clover	-	+	+	35±5.7	43.5±7.3*	-	+	+	58.7±22	51.4±18	-	+	+	36.6±7.4	35.7±10.4	-	+	+	41.3±6.1	39.9±3.4
	Subterranean clover	-	+	+	86.4±12.6	89.5±9	-	+	+	16.7±2.1	44.8±8.5*	-	+	+	30.9±3.7	32.4±1.8	+	+	+	23.9±4.9	49.7±11.4*
	Barrel medic	-	+	+	94.3±6.4	100	-	+	-	89±12.5	93.5±8.4	-	+	+	92.8±12	100	-	+	+	100	96.5±4.1
C.	Lentil	+	++	+	66±24	84.4±8.8	+	++	-	100	97.6±2.4	-	+	-	49.9±11.9	47±21.7	-	+	+	4.5±2.6	7.8±4
truncat	Soybean	+	+	+	6.40±1.3	9.48±2.8*	+	+	+	9.8±4.7	10.5±3	+	+	+	8.4±3.6	8±3.2	+	+	+	5±2.1	7.2±1.8
um	Faba bean	+	-	-	5.6±2.3	5.9±1.5	+	-	-	3.7±0.9	4.2±0.1	+	-	-	3.5±2	3.4±0.2	+	-	-	7.1±1.3	13.3±5.5*
isolate	Pea	+	-	-	2.1±1.1	1.9±0.6	-	+	-	0.98±0.4	0.9±0.4	-	+	-	6.2±1.1	5.4±1.6	-	+	-	1.2±0.9	3±1.1*
C431	Red clover	+	+	+	33.4±12.5	51.8±17*	-	+	-	56.6±17	61.9±14.7	-	+	+	41.5±14	85±15*	+	+	+	30.6±10.1	38.7±12.8
	White clover	+	+	+	27.6±3.9	33.2±5.8	-	+	-	43.9±7.5	91±8.3*	-	+	+	25±13	47±14.8*	-	+	+	18.1±8.6	29±7.8*
	Subterranean clover	-	+	+	51.3±8	54.1±18.2	-	+	-	90.8±10	98.1±3.3	-	+	+	57.4±16.3	92.4±11*	-	+	+	42.6±11.7	86.9±12.2*
	Barrel medic	-	+	+	93.1±8.1	92.7±5.4	-	+	-	45.9±14.2	44.1±2.3	-	+	-	47.8±11.9	43.6±0.9	+	+	+	92.1±7	90.2±5.7
C.	Lentil	-	++	+	35.4±14.4	95.6±4*	-	+	-	0.1±0.1	4.4±1.6	+	+	-	11.5±3.2	26.2±4.3*	-	+	-	4.7±0.9	15.2±2.8*
trifolii	Soybean	+	+	+	6.4±1.1	9.5±2.8*	+	+	+	5.4±1.8	5.8±2.3	-	+	+	7.2±3.3	6±2.5	+	-	-	13.2±3	10.2±0.7
C436	Faba bean	+	+	+	11.5±2.7	17.2±5.8	+	-	-	13.2±6.6	10.6±1.2	-	+	-	6.4±2.8	5.9±1.8	+	+	-	7.8±3.6	46.7±4.7*
	Pea	+	+	+	21±10.5	57±14.9*	+	-	-	2.4±0.9	2±0.4	+	-	-	1±0.5	2.5±0.9	+	-	-	3.2±1.4	9.6±3.9*
	Red clover	+	+	+	74.2±17.6	83.7±8.7	-	+	+	52.1±19	46.1±17	+	-	-	24.7±9.7	21.7±4.5	+	+	+	42.9±19.6	51.7±17

	White clover	+	+	+	53.3±5.6	85.3±16*	-	+	+	16.7±9.3	14±3.9	-	+	+	9±1.7	10.3±3	+	+	+	17.7±2.4	90±5.7*
	Subterranean clover	+	+	+	90.4±8.9	88±9.2	+	+	+	20±3.9	14±5.6	+	+	+	37.5±16	66±13*	+	+	+	51.8±12	90.6±9*
	Barrel medic	+	+	+	100	100	-	+	+	82.7±14.3	84.4±13.9	-	+	+	39.7±11.7	77.5±19*	+	+	+	45.7±8.1	42.1±2.6
CT	Untrated	-	-	-	0	0	-	-	-	0	0	-	-	-	0	0	-	-	-	0	0
	Water	-	-	-	0	0	-	-	-	0	0	-	-	-	0	0	-	-	-	0	0
	MeOH 5%	-	-	-	0	0	-	-	-	0	0	-	-	-	0	0	-	-	-	0	0

a:(N) necrotic leaf area, (DA) irregular discolored areas surrounded (or not) by a (NR) necrotic ring;

* %DS at 1 mg/ml and at 2 mg/ml is significantly different ($p < 0.05$)
